# Comparative efficacy of intravenous, topical, and combined tranexamic acid in elderly patients with intertrochanteric fractures undergoing intramedullary nail fixation: a multicenter cohort study

**DOI:** 10.3389/fphar.2026.1775124

**Published:** 2026-02-24

**Authors:** Qiushi Bai, Xiao Chang, Yuewang Li, YongMing Yang, Hongbo Zhang, Kunyu Ji, Xiongfei Zou, Baozhong Zhang

**Affiliations:** 1 Department of Orthopedics, Peking Union Medical College Hospital, Chinese Academy of Medical Sciences & Peking Union Medical College, Beijing, China; 2 Department of Orthopedics, Tuoketuo County Hospital, Inner Mongolia, China; 3 Department of Orthopedics, Ulanqab Traditional Chinese and Mongolian Medicine Hospital, Inner Mongolia, China; 4 Department of Orthopedics, Longfu Hospital, Beijing, China

**Keywords:** blood loss control, elderly patients, hip fracture surgery, intertrochanteric fracture, intramedullary nail fixation, tranexamic acid

## Abstract

**Background:**

Intertrochanteric fractures in older adults are associated with substantial blood loss, but the optimal tranexamic acid (TXA) regimen remains unclear. Most hip fracture studies have evaluated single-route intravenous TXA versus placebo, with limited data on combined regimens, particularly in the very old.

**Methods:**

We conducted a multicentre retrospective cohort study of patients aged ≥65 years with intertrochanteric fractures who underwent closed reduction and intramedullary nailing at four hospitals. Patients were classified into four groups by intraoperative TXA regimen: combined intravenous plus topical, intravenous only, topical only, or no TXA. Inverse probability weighting was used to address confounding and between-group imbalances. The primary outcome was total blood loss; intraoperative and hidden blood loss were also assessed. Secondary outcomes included transfusion status and volume and 90 day complications. Prespecified age strata (65–74, 75–84, and ≥85 years) were analysed for total blood loss and transfusion rate, and treatment-by-age interaction terms tested age-related differences in treatment effects.

**Results:**

We included 1,228 patients; weighting achieved good baseline balance between treatment groups. All TXA regimens significantly reduced total blood loss compared with no TXA, with the largest reduction in the intravenous-plus-topical group, which had the lowest transfusion rate and volume. TXA was not associated with higher rates of thrombotic events or other major complications, and postoperative length of stay was shorter in the intravenous-plus-topical and topical-only groups than in controls. Age-stratified analyses showed a significant treatment-by-age interaction for total blood loss; TXA reduced blood loss across all age strata, and its transfusion-sparing effect did not diminish with age.

**Conclusion:**

In older adults undergoing intramedullary fixation for intertrochanteric fractures, TXA reduces blood loss without increasing complications, with greatest benefit from combined intravenous plus topical use. These findings support TXA as a key component of perioperative blood management in this population.

## Introduction

Intertrochanteric fractures are common among older patients, typically resulting from low-energy trauma or falls. As the aging population grows, the incidence of these fractures continues to rise, and they are associated with substantial perioperative blood loss and a higher risk of postoperative complications (Tüzün et al., 2022). Closed reduction and internal fixation with a proximal femoral nail antirotation (PFNA) is a common surgical approach for treating intertrochanteric fractures, effectively stabilizing the fracture and promoting healing (Tang et al., 2023). However, perioperative blood loss, encompassing both intraoperative and hidden components, remains a significant clinical concern and contributes to increased transfusion requirements and postoperative complications such as thromboembolism and infection, which can adversely affect recovery (Cho et al., 2022).

Tranexamic acid (TXA), an antifibrinolytic agent, is widely used in orthopaedic surgery to reduce bleeding. By inhibiting fibrinolysis, TXA has been shown to reduce blood loss during procedures such as hip fracture surgery. TXA can be administered intravenously or topically (Dionne et al., 2022). Intravenous TXA rapidly enters the systemic circulation and reduces overall blood loss, but has raised concerns about potential systemic complications such as deep vein thrombosis (DVT) (Taeuber et al., 2021). Topical administration, on the other hand, delivers the drug directly to the surgical site, thereby reducing systemic exposure and potentially minimizing the risk of complications (Juraj et al., 2021).

While the effectiveness of TXA in reducing blood loss during hip fracture surgery is well established, direct comparisons among intraoperative TXA regimens remain limited, especially in older adults. In addition, it is unclear whether the effects of different regimens vary across age strata, with particular uncertainty in very old patients. To address these gaps, we conducted a multicenter retrospective study comparing perioperative outcomes among four TXA strategies, namely combined intravenous (IV) and topical TXA, IV TXA alone, topical TXA alone, and no TXA, in older adults undergoing PFNA for intertrochanteric fractures. We further explored potential effect heterogeneity across prespecified age strata.

## Methods

### Study design

This retrospective multicenter cohort study analyzed de-identified electronic health record (EHR) data from four hospitals in northern China (Hospitals A to D). Patients with intertrochanteric fractures who were admitted between 1 January 2018 and 31 December 2024 were included, and follow-up data were collected for up to 90 days after surgery. These hospitals serve as major referral centers for hip fracture management in the region and provide a large, regionally representative sample of patients. The study complied with the principles of the Declaration of Helsinki. Data retrieval and analysis followed strict confidentiality guidelines, and all personal identifiers were removed from the dataset.

### Participants

**FIGURE 1:**
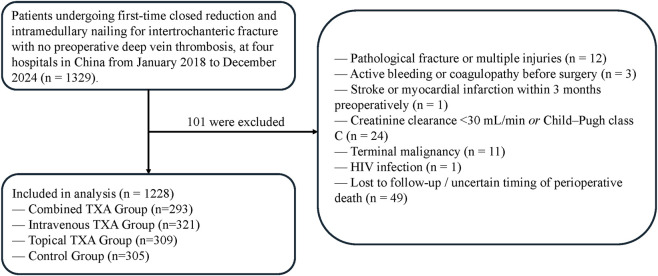
Flowchart of patient inclusion, exclusion, and group allocation.

Participants in this study were identified from the electronic health record systems of four hospitals in northern China. Patients were eligible if they were aged 65 years or older, had an intertrochanteric fracture confirmed by radiological assessment, underwent preoperative lower extremity Doppler ultrasonography that showed no deep vein thrombosis, and received closed reduction and intramedullary nail fixation during the index admission. Only patients with complete perioperative records were included. The diagnosis of intertrochanteric fracture was confirmed using admission and surgical codes together with radiology reports. Patients were excluded if they had pathologic fractures, multiple trauma, active bleeding, known coagulation disorders such as hemophilia, a history of cerebral infarction or myocardial infarction within 3 months before surgery, loss to follow-up during the perioperative period, terminal malignant disease, or human immunodeficiency virus infection. Participants were classified into four mutually exclusive groups according to the intraoperative TXA regimen ascertained from the EHR, including structured medication administration and operative notes. Intravenous TXA was identified by documented IV administration in the medication administration record, whereas topical TXA was identified by intraoperative documentation of TXA injection into the nail cavity. Patients were assigned to the combined group if both IV and topical TXA were documented, to the intravenous-only group if only IV TXA was documented, to the topical-only group if only topical TXA was documented, and to the control group if no intraoperative TXA administration was documented (Figure 1). Given the retrospective design and the use of routinely collected EHR data, no *a priori* sample size calculation was performed. During the study period, all eligible patients were consecutively identified from the EHR systems of each participating center, and no patients were excluded on the basis of TXA use. The choice of TXA regimen was determined by the treating team according to routine clinical practice, including center-specific surgeon preferences and patient-related considerations (e.g., anticipated thromboembolic risk or impaired renal function). Accordingly, complete withholding of TXA or use of topical TXA alone was part of routine care in selected patients. Therefore, the no-TXA group represents real-world management rather than an artificially constructed control. Consequently, the sample sizes across the four groups reflect real-world practice patterns across centers and over time.

### Procedures

Upon admission, all patients underwent routine clinical assessment and laboratory testing, including complete blood count and serum biochemistry. After comprehensive preoperative evaluation, patients were scheduled for closed reduction and intramedullary nail fixation within 48 h of admission under neuraxial or general anesthesia, as described in the Supplementary Methods sections on surgical procedure and perioperative fluid management. Perioperative red blood cell transfusion followed a standard protocol in accordance with Ministry of Health guidelines, in which transfusion was recommended when hemoglobin fell below 70 g/L or at higher levels when anemia-related organ dysfunction was suspected, for example, altered mental status, chest discomfort, palpitations or hypotension. Laboratory monitoring included complete blood counts on postoperative days 1–3, with additional testing performed when anemia was present or after transfusion. All patients received standard-dose low-molecular-weight heparin for prophylactic anticoagulation during the perioperative period. For TXA administration, intravenous TXA was given as a 1 g dose dissolved in 100 mL of saline and infused over 10 min during anesthesia induction (Zhang et al., 2022). For topical TXA, before insertion of the main intramedullary nail, tranexamic acid solution (1 g in 10 mL saline) was injected into the femoral medullary canal along the guidewire trajectory, and the procedure was paused for 2 min before continuing surgery, as previously described (Luo et al., 2025). Across participating centers, the topical TXA technique was standardized and confirmed with the lead operating surgeons at each hospital to be performed in the same manner. The combined treatment group received both intravenous and topical TXA, whereas the intravenous-only and topical-only groups received one route of administration. The control group did not receive TXA. Postoperative care followed standard protocols that included early mobilization to prevent complications such as deep vein thrombosis, and patients were monitored for adverse events potentially related to TXA, including thromboembolic events, as well as for other complications such as infection.

### Outcomes

The primary outcome of this study was total blood loss (TBL) during the perioperative period, which comprised intraoperative blood loss (IBL) and hidden blood loss (HBL). HBL was calculated using the Gross method, which estimates TBL and then derives HBL as the difference between TBL and IBL, as described in the Supplementary Methods section on HBL calculation. For blood loss calculations, hemoglobin (Hb) values were extracted from each center’s laboratory information system. The first recorded preoperative Hb value during the index admission was used as the baseline to ensure a consistent anchor across centers. Postoperative Hb measurements were obtained according to routine clinical practice (typically on postoperative days 1–3, with additional tests when clinically indicated). In patients who did not receive a transfusion, postoperative Hb was defined as the upward inflection point of Hb recovery within 7 days after surgery, which provides a standardized algorithm to accommodate variability in the timing of postoperative Hb testing across patients and centers. The secondary outcomes were perioperative blood transfusion status and perioperative transfusion volume, both of which were obtained from detailed entries in the electronic health record. We also recorded postoperative complications, including deep vein thrombosis, pulmonary embolism and wound infection. Clinical variables of interest included patient demographics such as age, sex and body mass index, comorbidities such as diabetes mellitus, hypertension and coronary artery disease, the American Society of Anesthesiologists (ASA) classification, preoperative hemoglobin and platelet levels, coagulation parameters including prothrombin time and activated partial thromboplastin time, surgical duration, anesthesia type, fracture classification based on the Evans-Jensen system and the time from injury to surgery. Postoperative follow-up was conducted at 30 and 90 days after surgery, and clinical complications were recorded at these time points (See supplementary document for complication assessment).

### Statistical analysis

Statistical analysis was performed using SPSS software (version 27.0). Descriptive statistics were used to summarize patient demographics and clinical characteristics. Continuous variables were presented as mean ± standard deviation or median with interquartile range, depending on the distribution of the data, and categorical variables were presented as counts and percentages. To mitigate baseline imbalances across treatment groups, inverse probability weighting (IPW) was applied, and all subsequent analyses were performed on the weighted cohort. Stabilized IPW were used, and the distribution of weights was examined to identify extreme values and ensure model robustness. A maximum standardized mean difference (Max |SMD|) of less than 0.1 was considered indicative of adequate balance of baseline characteristics between groups after weighting.

For continuous outcome variables, group comparisons were performed using one-way analysis of variance, and weighted multivariable linear regression models were fitted to estimate adjusted effects. For categorical outcome variables, group comparisons were performed using the chi-square test, and weighted multivariable logistic regression models were fitted. Model estimates and their corresponding confidence intervals were reported, and two-sided p values less than 0.05 were considered statistically significant.

Given the *a priori* hypothesis that the effect of TXA on blood loss might be modified by age, we conducted prespecified subgroup analyses of the primary outcome, dividing the population into three age groups (65–74, 75 to 84 and 85 years or older), corresponding to the young-old, old and oldest-old age strata proposed by the U.S. National Research Council Committee on Aging (Besdine, 1982). In addition, to formally test for effect modification by age, we included an interaction term between age group and treatment group in the weighted regression models. We then calculated weighted estimated marginal means (adjusted means) for each combination of age group and treatment group to facilitate interpretation of the interaction and to present model-adjusted differences between groups.

## Result

A total of 1,228 patients with intertrochanteric fractures were included and classified into four groups according to the intraoperative TXA regimen. The IV plus Top group received combined intravenous and topical TXA (n = 293), the IV group received intravenous TXA only (n = 321), the Top group received topical TXA only (n = 309), and the control group did not receive TXA (n = 305). Baseline demographic and clinical characteristics are summarized in Table 1. The four groups differed significantly in treatment center (P < 0.001), age (P < 0.001), prevalence of hypertension (P = 0.027), fracture classification (P = 0.027), and preoperative hemoglobin level (P < 0.001). The remaining variables were generally comparable, with no statistically significant differences. After application of IPW, baseline characteristics were well balanced across the groups (Supplementary Figure S1; Supplementary Table S1).

**TABLE 1:** Demographic and baseline characteristics.

Variable	Combined TXA group (n = 293)	Intravenous TXA group (n = 321)	Topical TXA group (n = 309)	Control group (n = 305)	P-value
Center	​	​	​	​	<0.001
A	96 (32.8)	86 (26.8)	62 (20.1)	61 (23.0)	​
B	71 (24.2)	72 (22.4)	79 (25.6)	76 (24.9)	​
C	61 (20.8)	80 (24.9)	103 (33.3)	69 (22.6)	​
D	65 (22.2)	83 (25.9)	65 (21.0)	99 (32.5)	​
Age, years (mean ± SD)	80.8 ± 5.7	77.4 ± 5.7	78.3 ± 5.7	78.3 ± 6.2	**<0.001**
Gender, n (%)	​	​	​	​	0.604
Male	117 (39.9)	126 (39.9)	111 (35.9)	125 (41.0)	—
Female	176 (60.1)	195 (60.7)	198 (64.1)	180 (59.0)	—
BMI, kg/m^2^ (mean ± SD)	24.4 ± 4.9	24.2 ± 5.1	23.9 ± 5.1	23.9 ± 5.0	0.603
Comorbidities, n (%)					
Diabetes	32 (10.9)	50 (15.6)	51 (16.5)	39 (12.8)	0.176
Hypertension	101 (34.5)	78 (24.3)	87 (28.2)	79 (25.6)	**0.027**
CHD	45 (15.4)	38 (11.8)	37 (12.0)	28 (9.2)	0.144
Fracture classification, n (%)	​	​	​	​	<0.001
Stable (I-II)	107 (36.5)	125 (38.9)	129 (41.7)	172 (56.4)	—
Unstable (III-V)	186 (63.5)	196 (61.1)	180 (58.3)	133 (43.6)	—
ASA classification, n (%)	​	​	​	​	0.064
II	80 (27.3)	88 (27.4)	92 (29.8)	85 (27.9)	—
III	193 (65.9)	227 (70.7)	197 (63.8)	207 (67.9)	—
IV	20 (6.8)	6 (1.9)	20 (1.6)	13 (4.3)	—
Anesthesia methods, n (%)	​	​	​	​	0.786
GA	107 (36.5)	123 (38.3)	108 (35.0)	117 (38.4)	​
EA	186 (63.5)	198 (61.7)	201 (65.0)	188 (61.6)	​
Preoperative laboratory tests					
Hgb, g/L (mean ± SD)	73.9 ± 7.7	79.3 ± 9.1	79.0 ± 9.2	83.3 ± 9.3	**<0.001**
PT, s (mean ± SD)	12.1 ± 1.4	12.1 ± 1.3	12.0 ± 1.4	12.1 ± 1.4	0.500
APTT, s (mean ± SD)	31.8 ± 3.2	32.2 ± 3.4	32.2 ± 3.3	32.0 ± 3.3	0.414
PLT, ×10^9^/L (mean ± SD)	133.7 ± 20.7	129.4 ± 22.0	130.9 ± 22.1	131.0 ± 22.5	0.113
Time from injury to surgery, days (mean ± SD)	3.7 ± 2.8	4.1 ± 3.3	3.9 ± 3.1	4.0 ± 2.9	0.592
Operation time, min (mean ± SD)	75.8 ± 28.5	76.2 ± 27.1	78.8 ± 28.6	75.8 ± 27.1	0.485

Bold values indicate statistical significance (P < 0.05).

Abbreviation: BMI, body mass index; CHD, coronary heart disease; ASA, american society of anesthesiologists; GA, general anesthesia; EA, epidural anesthesia; PT, prothrombin time; APTT, activated partial thromboplastin time; Hgb, Hemoglobin; PLT, platelet.

Table 2 summarizes perioperative blood loss and transfusion outcomes across the four treatment groups. Overall, TXA use was associated with significantly lower IBL, HBL and TBL compared with no TXA (all P < 0.001). The greatest reductions in TBL were observed in the IV + Top group, which had lower TBL than the control group and than either single route group, whereas TBL did not differ significantly between the IV and Top groups. Differences in TBL were largely driven by HBL. All TXA regimens reduced HBL compared with the control group, with the IV + Top group showing the lowest HBL and significantly lower HBL than both the IV and Top groups, and HBL in the Top group was also lower than in the IV group. In contrast, differences in IBL were smaller, and only the IV + Top and IV groups had significantly lower IBL than the control group, with no meaningful differences among the other pairwise comparisons (Supplementary Table S2). Transfusion outcomes showed a clear advantage only for the combined regimen. There were significant differences between groups in both transfusion rates and transfusion volumes (P < 0.001), and in pairwise comparisons the IV + Top group had lower transfusion rates and volumes than all other groups. The Top group had a modest reduction in transfusion volume compared with controls (P = 0.024), whereas transfusion rates did not differ significantly (P = 0.054), and other pairwise differences were not statistically significant (Supplementary Table S3).

**TABLE 2:** Weighted blood loss and transfusion rates across treatment groups after IPTW.

Variable	Combined TXA group	Intravenous TXA group	Topical TXA group	Control group	P-value
Intraoperative blood loss, mL (mean ± SD)	113.1 ± 77.0	114.5 ± 59.5	124.1 ± 90.5	135.9 ± 86.8	**0.001**
Hidden blood loss, mL (mean ± SD)	353.0 ± 77.8	401.0 ± 81.0	374.0 ± 91.5	438.0 ± 83.3	**<0.001**
Total blood loss, mL (mean ± SD)	466.2 ± 111.3	515.5 ± 103.0	498.0 ± 132.3	573.9 ± 120.6	**<0.001**
Perioperative transfusion rate (%)	139 (45.7)	182 (57.4)	169 (54.5)	188 (62.0)	**<0.001**
Perioperative transfusion volume, mL (mean ± SD)	134.6 ± 169.4	177.1 ± 185.2	171.7 ± 187.3	205.5 ± 198.2	**<0.001**

Bold values indicate statistical significance (P < 0.05).

Table 3 summarizes postoperative complications across the four treatment groups. There were no significant differences in the incidences of deep vein thrombosis, wound complications, postoperative pneumonia, 30 days mortality or 90 days mortality among the groups (all P > 0.05). In contrast, postoperative length of stay differed significantly between groups (P = 0.040). In pairwise comparisons, postoperative length of stay was shorter in the IV + Top group (P = 0.010) and the Top group (P = 0.016) than in the control group. In this cohort of older adults with intertrochanteric fractures, perioperative TXA use was not associated with a higher incidence of thrombotic events or other major postoperative complications.

**TABLE 3:** Postoperative complications across treatment groups.

Variable	Combined TXA group	Intravenous TXA group	Topical TXA group	Control group	P-value
Postoperative DVT, n (%)	21 (6.9)	33 (10.4)	21 (6.8)	22 (7.3)	0.275
Length of hospital stay (mean ± SD)	12.4 ± 6.3	13.0 ± 7.2	12.5 ± 6.6	13.9 ± 8.2	**0.040**
Pulmonary infection, n (%)	38 (12.5)	24 (7.6)	29 (9.4)	21 (6.9)	0.075
Poor wound healing, n (%)	14 (4.6)	18 (5.7)	20 (6.4)	10 (3.3)	0.311
30 day mortality, n (%)	7 (2.3)	16 (5.0)	13 (4.2)	11 (3.6)	0.340
90 day mortality, n (%)	20 (6.6)	32 (10.1)	21 (6.8)	25 (8.3)	0.334

Bold values indicate statistical significance (P < 0.05).

Patients were divided into three age subgroups (65–74 years, 75–84 years and 85 years or older). We first fitted a generalized linear regression model with TBL as the dependent variable. In this model, treatment group (P < 0.001), age group (P < 0.001), surgical time (P < 0.001) and the interaction between age group and treatment group (P = 0.009) were all significantly associated with TBL (Supplementary Table S4). To further describe TBL across treatment and age groups, we calculated estimated marginal means for TBL, which are presented in Table 4. These estimates suggested age related variation in TBL and in the absolute blood sparing effect of TXA. Although absolute blood loss increased with age, the blood sparing advantage of TXA, particularly in the intravenous plus topical treatment group, appeared to be maintained across all age groups. We did not find clear evidence that the treatment effect diminished with increasing age.

**TABLE 4:** Estimated marginal means of total blood loss by age group and treatment group (age group × treatment interaction).

Age group	Combined TXA group	Intravenous TXA group	Topical TXA group	Control group
65–74, mL [95% CI]	436.1 [406.6 to 465.5]	456.1 [429.8 to 482.3]	426.4 [396.6 to 456.3]	537.3 [510.3 to 564.3]
75–84, mL [95% CI]	459.6 [438.0 to 481.3]	520.0 [499.1 to 540.9]	494.6 [474.5 to 514.6]	557.0 [535.7 to 578.4]
>85, mL [95% CI]	488.1 [458.2 to 518.1]	563.7 [530.6 to 596.8]	572.5 [540.6 to 604.4]	636.0 [605.3 to 666.6]

Values are weighted estimated marginal means of total blood loss derived from a weighted general linear model including fixed effects for treatment group, age group, and their interaction (age group × treatment). The P value for the age group × treatment interaction was P = 0.009.

In addition, we fitted a logistic regression model with transfusion rate as the dependent variable. In this model, the IV + Top treatment group (P < 0.001) and the Top treatment group (P = 0.029) had significantly lower transfusion rates than the control group. Preoperative hemoglobin level (P < 0.001) and surgical time (P = 0.002) were also significantly associated with transfusion rate. The interaction between treatment group and age group was not statistically significant (Supplementary Table S5). To further describe transfusion rate across treatment groups and age groups, we calculated estimated marginal means of transfusion rate, which are presented in Table 5.

**TABLE 5:** Weighted estimated marginal means of blood transfusion rate by age group and treatment group (age group × treatment interaction).

Age group	Combined TXA group	Intravenous TXA group	Topical TXA group	Control group
65–74, % [95% CI]	37 [22 to 55]	42 [25 to 60]	50 [31 to 69]	74 [59 to 85]
75–84, % [95% CI]	43 [29 to 57]	66 [53 to 77]	57 [43 to 69]	74 [61 to 84]
>85, % [95% CI]	43 [25 to 63]	84 [64 to 94]	61 [39 to 79]	85 [70 to 93]

Values are weighted estimated marginal means of total blood loss derived from a weighted general linear model including fixed effects for treatment group, age group, and their interaction (age group × treatment). The P value for the age group × treatment interaction was P = 0.283.

## Discussion

In this multicenter retrospective cohort study of older adults undergoing intramedullary nail fixation for intertrochanteric fractures, we found that perioperative TXA use significantly reduced perioperative blood loss, transfusion rates and transfusion volumes, with combined intravenous and topical administration associated with the largest reductions. Across the entire cohort and within each age subgroup, patients treated with TXA had lower TBL than those who did not receive TXA, and the combined regimen produced the greatest reductions in both TBL and HBL (Zhou et al., 2019). In addition, TXA use was not associated with a higher incidence of venous thromboembolism or other major postoperative complications (Ghorbani et al., 2024), and combined intravenous and topical administration was associated with a shorter postoperative length of stay. These findings extend previous research by directly comparing three commonly used intraoperative TXA regimens with a no TXA group in a real world population of patients with intertrochanteric fractures treated with intramedullary nails. Overall, our results support a blood sparing benefit of TXA, particularly with combined intravenous and topical administration, and suggest that this benefit is preserved even in very old patients.

These findings are largely consistent with previous studies in hip fracture surgery, which have shown that TXA can reduce perioperative blood loss and generally decrease the need for transfusion (Lei et al., 2017). Prior studies focusing on intertrochanteric fractures have predominantly examined single route administration of TXA and have compared it with placebo or standard care. These studies typically report reduced blood loss without a clear increase in clinically relevant DVT (Liu and Lee, 2025). However, there is limited evidence directly comparing different TXA strategies in patients with intertrochanteric fractures treated with PFNA. In our study, combined intravenous and topical administration was more effective in reducing both TBL and HBL than either route alone, which is consistent with the hypothesis that systemic and local antifibrinolytic effects may provide complementary benefits (Yuan et al., 2018). In addition, combined intravenous and topical TXA reduced transfusion rates and volumes more than TXA given by either route alone (Haj-Younes et al., 2020). To our knowledge, previous studies have not systematically examined age as a modifier of the effects of different TXA regimens. Our results suggest that the blood sparing benefit of TXA is preserved across age strata, including very old patients, and that its ability to reduce perioperative transfusion rates does not appear to diminish with increasing age.

Based on the observed reduction in blood loss and transfusion volume, our findings are clinically significant and can be applied to perioperative blood management in elderly patients with intertrochanteric fractures. First, the consistent reduction in TBL and HBL with TXA supports its use as part of standard care pathways for intramedullary fixation, particularly in settings where perioperative anaemia and transfusion are common and associated with adverse outcomes. Stabilising haemoglobin levels may facilitate earlier mobilisation, reduce physiological stress and support smoother postoperative recovery (Lawrence et al., 2003). Second, the more favourable blood sparing profile of the IV plus Top regimen suggests that combined administration may be a reasonable option when the goal is to minimise blood loss, especially in patients with limited cardiopulmonary reserve or restricted access to blood products. Third, the preservation of TXA benefit across age strata indicates that advanced age alone should not preclude its use when standard thromboprophylaxis is provided. In the oldest patients, who often have the highest bleeding and transfusion risk, even modest reductions in blood loss may translate into clinically meaningful benefits (Willems et al., 2012). Clinicians should still individualise decisions, balancing bleeding and thrombotic risk, comorbidities and patient preferences, but our data suggest that TXA appears to be a generally safe and effective adjunct in this population.

Several limitations warrant consideration when interpreting these findings. The retrospective observational design means that the findings are subject to residual confounding and selection bias. Because TXA regimen selection was non-random, baseline imbalances could have biased unadjusted comparisons. Older age and lower preoperative hemoglobin are associated with a higher likelihood of transfusion and postoperative complications, and differences in fracture classification may reflect varying injury severity and surgical complexity, which can increase blood loss independent of TXA. These factors could therefore overestimate or underestimate regimen effects if higher-risk patients preferentially received (or were withheld) TXA. We used stabilized IPW and confirmed improved post-weighting balance, but residual confounding from unmeasured factors cannot be fully excluded; accordingly, our findings should be interpreted as associations. TXA use and choice of regimen were not randomised and instead reflected institutional protocols and surgeon preference, which may have been correlated with unmeasured factors such as perceived bleeding risk, frailty or intraoperative findings. Unmeasured clinician judgment and patient frailty may have influenced both regimen selection and the risks of transfusion and postoperative complications, resulting in confounding by indication that inverse probability weighting cannot fully eliminate. Although baseline demographic and clinical characteristics were broadly similar across groups, we cannot rule out the influence of unrecorded confounders, including subtle differences in surgical technique, intraoperative haemostasis or postoperative rehabilitation. Detection of venous thromboembolism and other complications relied on routine clinical assessment rather than systematic screening, so asymptomatic events may have been missed. This limitation could lead to underestimation of the true incidence of deep vein thrombosis and may reduce the power to detect modest differences in safety outcomes. In addition, the study was conducted in four hospitals within a single health system, and all patients underwent a specific surgical and anaesthetic approach, which may limit generalisability to settings with different perioperative protocols or patient populations. Finally, although the overall sample size was substantial, some subgroup analyses, particularly in the oldest age group and for infrequent complications, may still have been underpowered.

Despite these limitations, this multicentre cohort provides pragmatic evidence that TXA, particularly when administered as a combined intravenous and topical regimen, is associated with substantial reductions in perioperative blood loss in older adults undergoing intramedullary fixation of intertrochanteric fractures, without a clear increase in thromboembolic events or other major postoperative complications. Analyses stratified by age suggest that this blood sparing effect is preserved across older age groups, including patients aged 85 years and above. Future research should aim to confirm these findings in prospective or randomised studies that directly compare different TXA regimens, explore optimal dosing and timing and incorporate systematic assessment of venous thromboembolism and other adverse events. Trials that focus on very old patients at high risk, and that evaluate longer term outcomes such as functional recovery, quality of life, rehospitalisation and mortality, would be particularly informative. Long-term prognosis after proximal femur fracture remains poor. A 5 year follow-up study of patients aged 65 years and older reported 1 year and 5 year survival rates of 88.9% and 66.7%, with substantially lower survival among those aged 90 years and older, largely driven by comorbidity burden (Hashimoto et al., 2023). In addition, cost effectiveness analyses and implementation studies could help define how best to integrate TXA based blood management strategies into routine care for intertrochanteric hip fractures.

## Data Availability

The raw data supporting the conclusions of this article will be made available by the authors, without undue reservation.
