# Supplementary material for: Comparative efficacy of intravenous, topical, and combined tranexamic acid in elderly patients with intertrochanteric fractures undergoing intramedullary nail fixation: a multicenter cohort study
Source: Front Pharmacol. 2026 Feb 24;17:1775124. doi: 10.3389/fphar.2026.1775124 (PMC12971942; doi:10.3389/fphar.2026.1775124)
Supplement: Supplementary file 1 [file Supplementaryfile1.docx]

**Supplementary documents**

**Surgical procedure**

All procedures were performed by high-volume surgeons from the three centers using a standardized technique. In all cases, closed reduction and internal fixation were carried out with a proximal femoral nail antirotation system (PFNA-II; DePuy Synthes, Zuchwil, Switzerland) under fluoroscopic guidance. After induction of anesthesia, patients were positioned supine on a fracture/traction table with a padded perineal post; the injured limb was kept neutral and the contralateral limb abducted. Longitudinal traction with 15°–20° internal rotation was applied to obtain an anatomic or near-anatomic reduction on anteroposterior and lateral views. Through a 3–4-cm incision centered over the tip of the greater trochanter, the abductor fascia was bluntly split and the entry point exposed. A guidewire was advanced with the starting point at or slightly medial to the tip of the greater trochanter on the anteroposterior view and centered in the canal on the lateral view, followed by proximal femoral canal reaming and insertion of the nail. After confirming alignment, a helical blade was placed in a center–center (or inferior–center) position within the femoral head and seated approximately 5 mm short of the subchondral bone under biplanar fluoroscopy. The set screw was tightened to secure the blade, distal locking was performed according to fracture stability, and final fixation was verified (Figure S2).

**Perioperative fluid and hemodynamic management**

Perioperative fluid therapy followed a standardized conservative protocol across participating centers. Routine intravenous maintenance infusion was generally avoided unless one of the following conditions was present: (1) daily oral intake <1500 mL, in which case 5% dextrose solution was administered to maintain an approximately neutral fluid balance; or (2) clinical hypovolemia, in which case 500 mL of 6% hydroxyethyl starch 130/0.4 was infused. During surgery, isotonic saline was administered at a rate of 5 mL/kg/h to maintain intravascular volume and fluid balance. On the day of surgery, patients received a total of 1000 mL of 5% dextrose intravenously in accordance with the above protocol. Hemodynamic parameters were continuously monitored intraoperatively, and hypotension was managed at the discretion of the attending anesthesiologist with additional fluids and/or vasoactive agents as clinically indicated.

**Hidden blood loss (HBL) calculation formula**

The following formulae were used for calculation:

Blood volume (L) [1]:

Female: $({\frac{height (cm)}{100})}^{3}\times0.356+weight (kg)\times0.033+0.183$

Male: $({\frac{\mathrm{height}\left( \mathrm{cm} \right)}{100})}^{3}\times0.367+weight (kg)\times0.032+0.604$

Estimated blood loss (EBL) (g) (assuming 55 g of haemoglobin in 1 unit and the same concentration of haemoglobin in each blood product) [2]:

$$Blood volume (L) \times\left( \mathrm{Hgb}_{\mathrm{pre}} (g/L)- \mathrm{Hgb}_{\mathrm{post}} (g/L) \right)+\frac{IBTV(mL)-PBTV(mL)}{200}\times55$$

EBL (mL):

$$\frac{EBL volume (g)}{\mathrm{HGB}_{\mathrm{pre}} (g/L)}\times1000$$

HBL (mL):

$$EBL volume (mL) - Intraoperative blood loss (mL)$$

IBTV, intraoperative blood transfusion volume; PBTV, Postoperative blood transfusion volume; Hgb, haemoglobin

**Complication assessment**

Postoperative complications were assessed using a prespecified surveillance strategy and standardized definitions across centers. Venous thromboembolism (VTE) (deep vein thrombosis [DVT] and pulmonary embolism) was evaluated over the 90-day postoperative period. At admission, all patients underwent baseline bilateral lower-limb duplex ultrasonography to exclude pre-existing DVT. During hospitalization, clinicians performed daily bedside assessments for clinical signs and symptoms suggestive of DVT, and a routine bilateral duplex scan on postoperative day 3 was conducted by senior sonographers. Within 90 days after surgery, patients who reported symptoms suggestive of DVT at scheduled follow-up or during unplanned visits underwent confirmatory testing as clinically indicated, including repeat duplex ultrasonography, CT pulmonary angiography, or contrast venography. All thromboembolic events and other adverse events occurring within 90 days postoperatively were captured from the EHR and verified against imaging reports and discharge summaries. In addition, early postoperative complications, specifically poor wound healing and pulmonary infection, were assessed as events occurring within 30 days after surgery, based on inpatient records, follow-up documentation, and relevant diagnostic information recorded in the EHR.

**Reference**

1. Nadler SB, Hidalgo JH, Bloch T. Prediction of blood volume in normal human adults. Surgery. 1962; 51(2): 224-32.

2. Gross JB. Estimating allowable blood loss: corrected for dilution. Anesthesiology. 1983; 58(3): 277-80.

**Figure Legend**

**Figure S1.** Standardized mean differences of baseline covariates before and after inverse probability weighting. The vertical reference line at 10 percent represents the threshold for acceptable balance, with values closer to zero indicating better balance between groups.

**Figure S2.** Representative anteroposterior radiographs of intertrochanteric fracture before and after intramedullary nail fixation. (A) Preoperative anteroposterior X-ray demonstrating a typical intertrochanteric fracture pattern. (B) Postoperative anteroposterior X-ray obtained after intramedullary nail fixation showing satisfactory reduction and stable implant position.

**Table S1.** Baseline Characteristics Across TXA Treatment Groups After IPTW Weighting

| **Variable** | Combined TXA Group | Intravenous TXA Group | Topical TXA Group | Control Group | Max \|SMD\| |
| --- | --- | --- | --- | --- | --- |
| **Center** |  |  |  |  | 0.100 |
| **A** | 67 (22.0) | 77 (24.3) | 79 (255) | 73 (24.2) |  |
| **B** | 86 (28.2) | 81 (25.6) | 74 (23.9) | 72 (23.8) |  |
| **C** | 75 (24.6) | 80 (25.2) | 79 (25.5) | 82 (27.2) |  |
| **D** | 77 (25.2) | 79 (24.9) | 78 (25.2) | 75 (24.8) |  |
| Age, years (mean ± SD) | 78.9 ± 6.2 | 78.7 ± 6.0 | 78.7 ± 5.7 | 78.7 ± 6.1 | 0.035 |
| Gender, n (%) |  |  |  |  | 0.099 |
| Male | 129 (42.3) | 132 (41.6) | 119 (38.3) | 113 (37.3) |  |
| Female | 176 (57.7) | 185 (58.4) | 192 (61.7) | 190 (62.7) |  |
| BMI, kg/m^2^ (mean ± SD) | 24.2 ± 5.0 | 24.1 ± 5.0 | 24.2 ± 5.1 | 24.0 ± 5.1 | 0.046 |
| Comorbidities, n (%) |  |  |  |  |  |
| Diabetes | 51 (16.8) | 43 (13.6) | 42 (13.5) | 45 (14.9) | 0.088 |
| Hypertension | 82 (27.0) | 90 (28.4) | 86 (27.7) | 86 (28.5) | 0.025 |
| CHD | 39 (12.8) | 38 (12.0) | 39 (12.6) | 36 (11.9) | 0.033 |
| Fracture Classification, n (%) |  |  |  |  | 0.025 |
| Stable (Ⅰ-Ⅱ) | 132 (43.4) | 135 (42.6) | 134 (43.1) | 128 (42.2) | - |
| Unstable (Ⅲ-Ⅴ) | 172 (56.6) | 182 (57.4) | 177 (56.9) | 175 (57.8) | - |
| ASA Classification, n (%) |  |  |  |  | 0.063 |
| Ⅱ | 89 (29.3) | 91 (28.7) | 86 (27.7) | 81 (26.7) | - |
| Ⅲ | 201 (66.1) | 214 (67.5) | 208 (67.1) | 207 (68.3) | - |
| Ⅳ | 14 (4.6) | 12 (3.8) | 16 (5.2) | 15 (5.0) | - |
| Anesthesia Methods, n (%) |  |  |  |  | 0.101 |
| GA | 104 (34.2) | 114 (36.0) | 117 (37.7) | 118 (39.1) |  |
| EA | 200 (65.8) | 203 (64.0) | 193 (62.3) | 184 (60.9) |  |
| Preoperative laboratory tests |  |  |  |  |  |
| Hgb, g/L (mean ± SD) | 79.5 ± 9.8 | 78.7 ± 9.4 | 78.7 ± 9.4 | 79.2 ± 9.1 | 0.090 |
| PT, s (mean ± SD) | 12.1 ± 1.5 | 12.1 ± 1.3 | 12.1 ± 1.4 | 12.0 ± 1.3 | 0.126 |
| APTT, s (mean ± SD) | 32.3 ± 3.1 | 32.0 ± 3.4 | 32.0 ± 3.4 | 32.1 ± 3.3 | 0.085 |
| Plt, ×10^9^/L (mean ± SD) | 132.1 ± 21.4 | 130.7 ± 22.8 | 131.4 ± 21.8 | 131.1 ± 22.6 | 0.062 |
| Time from Injury to Surgery, days (mean ± SD) | 4.3 ± 3.2 | 3.9 ± 3.2 | 4.1 ± 3.3 | 3.9 ± 2.7 | 0.103 |
| Operation Time, min (mean ± SD) | 76.6 ± 30.5 | 77.1 ± 28.0 | 76.5 ± 27.3 | 78.1 ± 26.5 | 0.060 |

BMI, Body Mass Index; CHD, Coronary heart disease; ASA, American Society of Anesthesiologists; GA, General Anesthesia; EA, Epidural Anesthesia; PT, Prothrombin Time; APTT, Activated Partial Thromboplastin Time; Hgb, Hemoglobin; Plt, Platelet.

**Table S2.** Comparison of Blood Loss Across Treatment Groups and 95% Confidence Intervals

| Comparison Group | Difference | 95% CI | P-value |
| --- | --- | --- | --- |
| IBL |  |  |  |
| A vs B | -1.3 | -13.8 to 11.2 | 0.837 |
| A vs C | -10.9 | -23.5 to 1.6 | 0.088 |
| A vs D | -22.8 | -35.4 to -10.1 | **<0.001** |
| B vs C | -9.6 | -22.0 to 2.8 | 0.128 |
| B vs D | -21.5 | -33.9 to -9.0 | **<0.001** |
| C vs D | -11.8 | -24.4 to 0.7 | 0.065 |
| HBL |  |  |  |
| A vs B | -48.0 | -61.2 to -34.9 | **<0.001** |
| A vs C | -20.9 | -34.2 to -7.7 | 0.002 |
| A vs D | -85.0 | -98.3 to -71.7 | **<0.001** |
| B vs C | 27.1 | 14.0 to 40.2 | **<0.001** |
| B vs D | -37.0 | -50.1 to -23.8 | **<0.001** |
| C vs D | -64.0 | -77.3 to -50.8 | **<0.001** |
| TBL |  |  |  |
| A vs B | -49.3 | -67.8 to -30.9 | **<0.001** |
| A vs C | -31.7 | -50.4 to -13.3 | **<0.001** |
| A vs D | -107.7 | -126.4 to -89.1 | **<0.001** |
| B vs C | 17.5 | -0.9 to 35.8 | 0.062 |
| B vs D | -58.4 | -76.9 to -39.9 | **<0.001** |
| C vs D | -75.9 | -94.5 to -57.3 | **<0.001** |

Abbreviation: Group A, Combined TXA Group; Group B, Intravenous TXA Group; Group C, Topical TXA Group; Group D, Control Group;

**Table S3.** Comparison of Blood Transfusion Across Treatment Groups and Relative Risk

| Comparison Group | Difference (%) | 95% CI (%) | P-value | RR | 95% CI | P-value |
| --- | --- | --- | --- | --- | --- | --- |
| Transfusion Rates |  |  |  |  |  |  |
| A vs B | -11.8 | -19.6 to -3.9 | **0.003** | 0.79 | 0.68 to 0.93 | **0.004** |
| A vs C | -8.9 | -16.7 to -1.0 | **0.027** | 0.84 | 0.71 to 0.98 | **0.029** |
| A vs D | -16.5 | -24.4 to -8.7 | **<0.001** | 0.73 | 0.63 to 0.85 | **<0.001** |
| B vs C | 2.9 | -4.8 to 10.7 | 0.459 | 1.05 | 0.92 to 1.21 | 0.459 |
| B vs D | -4.7 | -12.4 to 3.0 | 0.229 | 0.92 | 0.81 to 1.05 | 0.230 |
| C vs D | -7.7 | -15.5 to 0.1 | 0.054 | 0.88 | 0.77 to 1.00 | 0.055 |
| Transfusion Volume |  |  |  |  |  |  |
| A vs B | -42.6 | -71.8 to -13.4 | **0.004** | - | - | - |
| A vs C | -37.1 | -66.5 to -7.8 | **0.013** | - | - | - |
| A vs D | -70.9 | -100.4 to -41.4 | **<0.001** | - | - | - |
| B vs C | 5.4 | -23.6 to 34.5 | 0.713 | - | - | - |
| B vs D | -28.4 | -57.6 to 0.9 | 0.057 | - | - | - |
| C vs D | -33.8 | -63.2 to -4.4 | **0.024** | - | - | - |

Abbreviation: Group A, Combined TXA Group; Group B, Intravenous TXA Group; Group C, Topical TXA Group; Group D, Control Group;

**Table S4.** Statistical Analysis of Total Blood Loss By General Linear Model

| Source | Type Ⅲ sum of Squares | df | Mean Square | P-value |
| --- | --- | --- | --- | --- |
| Corrected model | 4693094.75 | 29 | 161830.85 | **<0.001** |
| Intercept | 736312.23 | 1 | 736312.23 | **<0.001** |
| **Treatment** | 1834105.83 | 3 | 611368.61 | **<0.001** |
| **Center** | 37642.99 | 3 | 12547.67 | 0.382 |
| Age group | 1178326.85 | 3 | 589163.42 | **<0.001** |
| Gender | 1803.49 | 1 | 1803.49 | 0.867 |
| BMI | 20.22 | 1 | 20.22 | 0.910 |
| Diabetes | 346.87 | 1 | 346.87 | 0.398 |
| Hypertension | 156.25 | 1 | 156.25 | 0.205 |
| CHD | 8759.60 | 1 | 8759.60 | 0.628 |
| Fracture Classification | 2879.74 | 1 | 2879.74 | 0.702 |
| ASA Classification | 38957.66 | 2 | 19478.83 | 0.815 |
| Anesthesia methods | 674.20 | 1 | 674.20 | 0.347 |
| Preoperative Hgb | 10868.65 | 1 | 10868.65 | 0.968 |
| Preoperative PT | 8497.66 | 1 | 8497.66 | 0.405 |
| Preoperative APTT | 3841.54 | 1 | 3841.54 | 0.576 |
| Preoperative PLT | 298.76 | 1 | 298.76 | 0.876 |
| Time from Injury to Surgery | 270.29 | 1 | 270.29 | 0.882 |
| Operation Time | 892279.41 | 1 | 892279.41 | **<0.001** |
| **Treatment *** Age group | 212142.03 | 6 | 35357.00 | **0.009** |

**Table S5.** Factors Associated with Transfusion Based on Binary Logistic Regression Model

| Source | B | SE | 95%CI | P-value |
| --- | --- | --- | --- | --- |
| Intercept | 24.03 | 2.18 | 19.76 to 28.30 | **<0.001** |
| **Treatment =** IV + Top group | -2.038 | 0.57 | -3.16 to -0.92 | **<0.001** |
| **Treatment =** IV group | -0.09 | 0.67 | -1.39 to 1.22 | 0.894 |
| **Treatment =** Top group | -1.32 | 0.61 | -2.51 to -0.13 | **0.029** |
| **Center = A** | -0.27 | 0.25 | -0.75 to 0.21 | 0.272 |
| **Center = B** | -0.15 | 0.24 | -0.62 to 0.32 | 0.536 |
| **Center = C** | 0.25 | 0.24 | -0.71 to 0.21 | 0.292 |
| Age Group = 65-74 | -0.69 | 0.53 | -1.73 to 0.34 | 0.188 |
| Age Group = 75-84 | -0.71 | 0.49 | -1.67 to 0.24 | 0.144 |
| Gender | 0.16 | 0.30 | -0.43 to 0.75 | 0.594 |
| BMI | 0.00 | 0.02 | -0.03 to 0.03 | 0.934 |
| Diabetes | 0.24 | 0.24 | -0.23 to 0.71 | 0.310 |
| Hypertension | 0.21 | 0.21 | -0.20 to 0.62 | 0.310 |
| CHD | 0.24 | 0.25 | -0.26 to 0.73 | 0.344 |
| Fracture Classification | -0.01 | 0.18 | -0.36 to 0.33 | 0.947 |
| ASA Classification = Ⅱ | 0.42 | 0.43 | -0.43 to 1.27 | 0.335 |
| ASA Classification = Ⅲ | 0.33 | 0.41 | -0.47 to 1.14 | 0.418 |
| Anesthesia methods | -0.09 | 0.17 | -0.43 to 0.25 | 0.610 |
| Preoperative Hgb | -0.29 | 0.16 | -0.32 to -0.26 | **<0.001** |
| Preoperative PT | -0.05 | 0.11 | -0.26 to 0.16 | 0.650 |
| Preoperative APTT | 0.01 | 0.03 | -0.04 to 0.06 | 0.662 |
| Preoperative PLT | -0.00 | 0.00 | -0.01 to 0.01 | 0.725 |
| Time from Injury to Surgery | -0.04 | 0.03 | -0.09 to 0.01 | 0.114 |
| Operation Time | 0.01 | 0.00 | 0.00 to 0.01 | **0.002** |
| **[Treatment =** IV + Top group**]** * [Age Group = 65-74] | 0.45 | 0.71 | -0.95 to 1.84 | 0.531 |
| **[Treatment =** IV + Top group**]** * [Age Group = 75-84] | 0.70 | 0.66 | -0.59 to 1.99 | 0.286 |
| **[Treatment =** IV group**]** * [Age Group = 65-74] | -1.31 | 0.80 | -2.88 to 0.25 | 0.100 |
| **[Treatment =** IV group**]** * [Age Group = 75-84] | -0.29 | 0.73 | -1.72 to 1.15 | 0.693 |
| **[Treatment =** Top group**]** * [Age Group = 65-74] | 0.25 | 0.75 | -1.22 to 1.72 | 0.737 |
| **[Treatment =** Top group**]** * [Age Group = 75-84] | 0.55 | 0.68 | -0.78 to 1.87 | 0.418 |
