# Supplementary figures and images for: Comparative efficacy of intravenous, topical, and combined tranexamic acid in elderly patients with intertrochanteric fractures undergoing intramedullary nail fixation: a multicenter cohort study
Source: Front Pharmacol. 2026 Feb 24;17:1775124. doi: 10.3389/fphar.2026.1775124 (PMC12971942; doi:10.3389/fphar.2026.1775124)

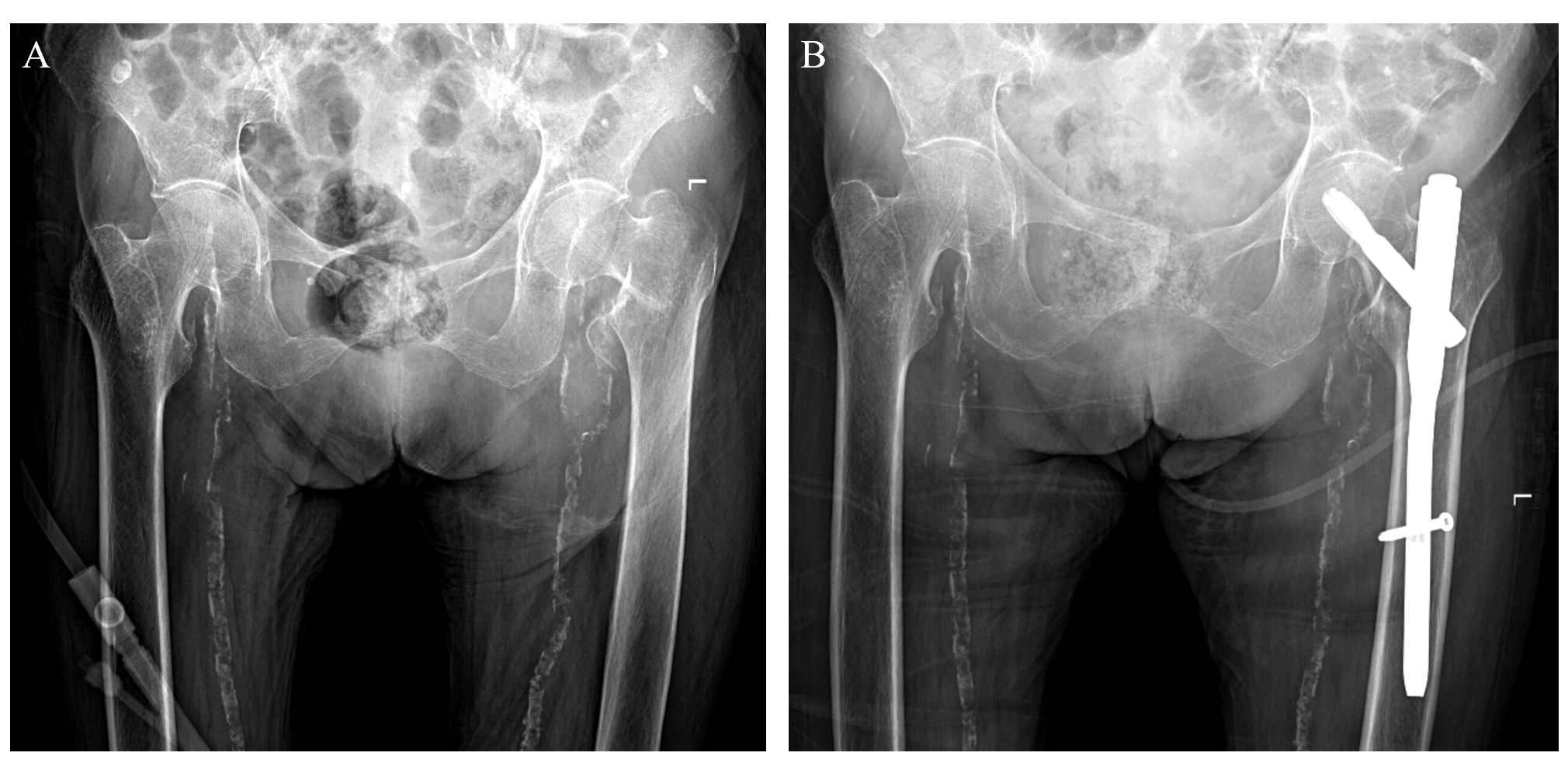

Supplement: Supplementary file 2 [file Image2.jpeg]
